# Development of a flow chamber system for the reproducible *in vitro* analysis of biofilm formation on implant materials

**DOI:** 10.1371/journal.pone.0172095

**Published:** 2017-02-10

**Authors:** Henryke Rath, Sascha Nico Stumpp, Meike Stiesch

**Affiliations:** Department for Prosthetic Dentistry and Biomedical Materials Science, Hannover Medical School, Hannover, Germany; Virginia Commonwealth University, UNITED STATES

## Abstract

Since the introduction of modern dental implants in the 1980s, the number of inserted implants has steadily increased. Implant systems have become more sophisticated and have enormously enhanced patients’ quality of life. Although there has been tremendous development in implant materials and clinical methods, bacterial infections are still one of the major causes of implant failure. These infections involve the formation of sessile microbial communities, called biofilms. Biofilms possess unique physical and biochemical properties and are hard to treat conventionally. There is a great demand for innovative methods to functionalize surfaces antibacterially, which could be used as the basis of new implant technologies. Present, there are few test systems to evaluate bacterial growth on these surfaces under physiological flow conditions. We developed a flow chamber model optimized for the assessment of dental implant materials. As a result it could be shown that biofilms of the five important oral bacteria *Streptococcus gordonii*, *Streptococcus oralis*, *Streptococcus salivarius*, *Porphyromonas gingivalis*, and *Aggregatibacter actinomycetemcomitans*, can be reproducibly formed on the surface of titanium, a frequent implant material. This system can be run automatically in combination with an appropriate microscopic device and is a promising approach for testing the antibacterial effect of innovative dental materials.

## Introduction

More than 700 bacterial species inhabit the human oral cavity [1]. While a large proportion of these bacteria are harmless commensals, opportunistic bacteria can trigger common oral diseases like caries, peri-implantitis and the chronic inflammatory disease periodontitis [1, 2]. Most bacteria within the oral cavity are sessile and form highly organised microbial communities, referred to as biofilm, on the surfaces of soft and hard tissues. Bacteria are embedded in a matrix of self-secreted extracellular polymeric substances (EPS) that determine the three dimensional structure of the biofilm [3]. As the EPS matrix shields cells in the biofilm from antimicrobials as well as from the host immune response, sessile bacteria can exhibit up to 5000-fold greater resistance to antibiotics than free floating (planktonic) cells [4]. There are three mechanisms that increase antibiotic resistance: First, the EPS acts as a potent diffusion barrier that antagonizes antibiotic penetration into the biofilm [5, 6]. Second, the sessile life cycle stage is accompanied by a metabolic activity change that reduces the antibiotic uptake [7–11]. Third, via horizontal gene transfer resistance genes can be exchanged between bacteria in biofilms as reported by Roberts et al. [12]. The high antibiotic tolerance makes biofilm infections hard to treat. In spite of intensive research, these bacterial communities still pose a severe medical complication. Therefore, the biofilm-related infections belong to the main reasons for early and late implant loss in dental implantology, as well as in other medical disciplines [13]. Therefore, studies are necessary investigating oral biofilm formation in order to develop implant surfaces that reduce biofilm formation and the risk of implant failure.

Even tough flow chamber systems have been used to study biofilm formation in oral implants research, most studies were conducted under static conditions [14–18]. These require a less sophisticated experimental set-up and show greater ease of handling [19]. However, the physiological flow conditions, as they are found at the dental implantation site, are not considered in these systems. Even though, the application of a static or dynamic system depends on the considered scientific question. The microenvironmental conditions have substantial impact on biofilm morphology and growth behavior as they influence: a) oxygen and nutrients transport processes through the biofilm [20–22], b) transport of signal molecules, e.g. quorum sensing messengers that alternates the biofilm morphology and physiology [23–25], c) heat and mass transfer that coordinates the biotransformation reaction and energy losses [21], d) gene expression and EPS content [26] and e) spreading of biofilms through increasing the mobility of pioneering bacterial cells [26].

Sternberg et al., Besemer et al., Purevdori et al., and Zhang et al. focused on a variety of features of biofilms, including the influence of flow velocity on biofilm morphology and the distribution of bacterial growth activity within a flow chamber system [23, 27–30]. Biofilm behavior was also tested under different chemical and physiological conditions as the pH or the different nutrient and oxygen concentrations in a flow environment [20, 21, 23, 27–30].

Clinical research with flow chamber systems has increased rapidly within the last decade. For example, Zhao et al. developed a model to investigate different implant materials for the suppression of biofilm formation and Chin et al. used a flow chamber system to test the effects of antibacterial agents on orthodontic binding materials [31, 32]. In 2013, da Silva Domingues et al. developed a parallel flow chamber system to elucidate the adherence mechanisms of *Staphylococcus aureus* and quantify phagocytosis by murine and human macrophages [33]. Even though, dynamic studies of oral biofilm formation and characterization have to be improved. These studies have to include the implant material, implant failure relevant bacteria, reproducible culture and flow conditions.

Biofilm architecture is greatly influenced by the colonising bacterial species or the species composition. The present study focused on a selection of oral biofilm formers that reside within the oral cavity during health and/or disease: *S*. *gordonii*, *S*. *oralis*, *S*. *salivarius*, *P*. *ginigivalis*, and *A*. *actinomycetemcomitan*s. The gram-positive streptococci *S*. *gordonii*, *S*. *oralis* and *S*. *salivarius* are commensal bacteria that, as pioneer colonisers, provide adhesion sites to middle and late colonizing bacterial species [34, 35]. *S*. *salivarius* is an opportunistic pathogen that may occasionally cause infections such as caries in man and which is involved in the development of halitosis [36, 37]. One of the most virulent opportunistic pathogenic streptococci is *S*. *oralis*. This bacterium expresses a sialidase, an enzyme that hydrolyses the bonds between sialic acids residues and the hexose or hexosamine residues at the terminal side of the oligosaccharides in glycolipids and glycoproteins. In the bacterial host, the enzyme cleaves target structures and thus unfavourably influences cellular processes [38–41]. Moreover, *S*. *oralis* causes infective endocarditis and is a major pathogen in immunosuppressed patients [42–45].

*P*. *gingivalis* and *A*. *actinomycetemcomitan*s are rod-shaped, anaerobic, gram-negative bacteria that can cause severe diseases, including periodontitis [46–48].

*P*. *gingivalis* secretes inflammatory compounds and toxins that attack host tissue and can lead dysbiosis of the microbial flora [49]. *A*. *actinomycetemcomitan*s produces virulence factors that allow the migration and invasion of other bacteria, e.g. the expression of leukotoxins. As a result, host periodontal tissue is damaged and the immune response severely weakened [50], [51].

For oral implant materials testing a standardized *in vitro* biofilm model for a panel of relevant bacterial species is missing. The aim of the study was to establish and evaluate a flow chamber system for this purpose. The experimental parameters growth environment, cultivation duration and nutrient supply had to be optimized for the biofilm formers, *S*. *gordonii*, *S*. *oralis*, *S*. *salivarius*, *P*. *gingivalis* and *A*. *actinomycetemcomitan*s to give a reproducible and robust biofilm formation *in vitro*.

## Materials and methods

### Bacterial strains

*S*. *gordonii* DSM 20568, *S*. *salivarius* DSM 20067 and *P*. *gingivalis* DSM 20709 were obtained from the German Collection of Microorganisms and Cell Cultures (DSMZ). The bacterial strains *S*. *oralis* ATCC 9811 and *A*. *actinomycetemcomitans* ATCC 2474 were purchased from the American Type Culture Collection (ATCC).

### Bacterial cultivation and biofilm formation

*S*. *gordonii*, *S*. *salivarius* and *S*. *oralis* were routinely precultured in Tryptic Soy Broth (TSB; Oxoid, Unipath Ltd., Wesel, Germany), supplemented with 10% yeast extract (Roth, Karlsruhe, Germany), aerobically at 37°C in an incubator shaker (200 min^-1^, SM-30, Bühler, Utzwil, Switzerland) for 18 h (*S*. *gordonii*, *S*. *salivarius*) or 24 h (*S*. *oralis*). For the induction of biofilm formation, *S*. *gordonii* and *S*. *salivarius* cultures were adjusted to an optical density (OD_600_; BioPhotometer, Eppendorf, Hamburg, Germany) of 0.016 in TSB medium modified by addition of 50 mM glucose (Roth) and stirred (VMS-C7 advanced, VWR; Darmstadt, Germany) for 24 h at 37°C for biofilm formation. This OD_600_ corresponds to an inoculum of: 1.94 x 10^6^ colony forming units CFU/mL for *S*. *gordonii* and 4.19 x 10^6^ CFU/mL for *S*. *salivarius*, respectively. For *S*. *oralis*, the procedure was identical, except that an OD_600_ of 0.026 was chosen (3.5 x 10^6^ CFU/mL) and that Brain Heart Infusion (BHI, Oxoid, Unipath Ltd., Wesel, Germany) medium supplemented with 5% sucrose (Roth) and 10 μg/mL vitamin K (Roth) was used as nutrient broth. *S*. *oralis* biofilm was also cultivated for 24 h.

*P*. *gingivalis* was cultured anaerobically (10% carbon dioxide, 10% hydrogen, 80% nitrogen) in BHI medium modified with 10 μg/mL of vitamin K for 24 h without agitation at 37°C, followed by culture in an incubator shaker (200 min^-1^) for 48 h. For biofilm formation experiments, the cultures were adjusted to an OD_600_ of 0.0375 (7.88 x 10^6^ CFU/mL) in BHI medium supplemented with 5% sucrose and 10 μg/mL vitamin K. The bacterial suspension was anaerobically grown under continuous stirring for 48 h at 37°C for biofilm formation.

*A*. *actinomycetemcomitans* was routinely cultured anaerobically in Schaedler bouillon (Oxoid, Unipath Ltd., Wesel, Germany) supplemented with 10 μg/mL vitamin K at 37°C under agitation (200 min^-1^) for 48 h and was then transferred to an Erlenmeyer flask to be cultured for an additional 48 h under continuous stirring. To induce biofilm formation by *A*. *actinomycetemcomitans*, cultures were adjusted to an OD_600_ of 0.0319 (1.25 x10^6^ CFU/mL) in Schaedler bouillon supplemented with 10 μg/mL vitamin K and anaerobically cultured for 72 h at 37°C under continuous stirring for biofilm formation. All experiments modified with vitamin K were protected from light to avoid the destruction of the compound by light. For the anaerobic cultivation of *P*. *gingivalis* and *A*. *actinomycetemcomitans*, the bioreactor and the flow chamber system were evacuated twice and subsequently flushed with anaerobic gas mix (10% hydrogen, 10% carbon dioxide, 80% nitrogen). The system was kept pressurized by connecting a gas filled balloon to the bioreactor. Thus the inflow of oxygen was inhibited throughout the experiment.

### Flow chamber system

**Fig 1:**
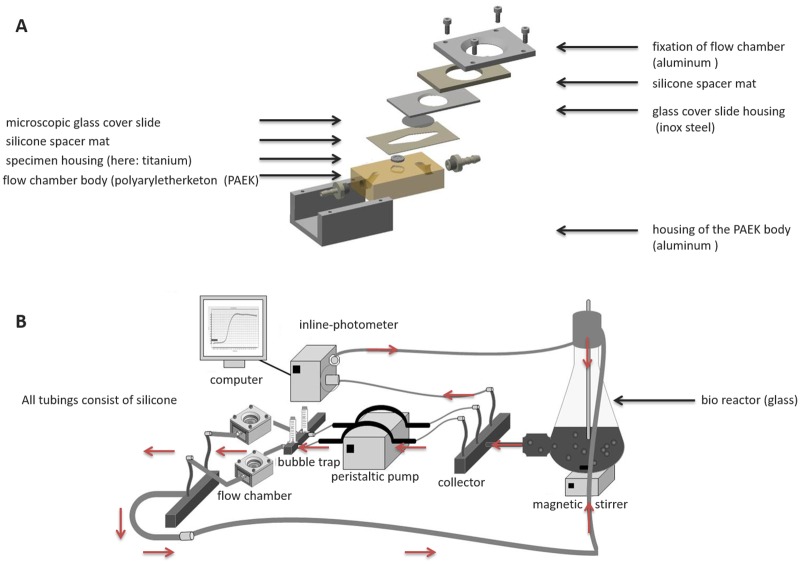
Sketch of the flow chamber and flow chamber system. A) Assembly drawing with test specimen; B) General setup of the closed circuit system. The red arrows indicate the direction of the flow.

The flow chamber devices were 7.0 cm x 5.5 cm x 3.5 cm in size and were equipped with a 28 mm glass cover slip (Thermo Scientific, Waltham, USA) to allow direct macro- and microscopic analysis of biofilm formation on test surfaces (Fig 1). Titanium discs (grade 4) with a diameter of 12 mm were used as specimens. Each of these underwent surface treatment with a 45 μm diamond abrasives grinding disc to generate a uniform surface pattern for bacterial adhesion. The bacterial suspension was recirculated from the bioreactor to the flow chambers and back at a flow rate of 100 μL/min (IPC-16 peristaltic pump, Ismatec, Wertheim, Germany). The flow chamber system was kept air-free by use of bubble traps. To monitor bacterial growth in the bioreactor, the OD_600_ was continuously recorded during the experiment using an inline photometer (Elo-Check, biotronix, Hennigsdorf, Germany). Each flow chamber experiment was repeated independently five times with at least three technical replicates.

### Biofilm imaging and analysis

The flow chambers were separated from the system by surgical clamps at the collector’s site. New sterile tubings were connected to the tubings of the peristaltic pump. Biofilms were washed by pumping phosphate buffered saline (PBS, Dulbecco`s Media, Sigma Aldrich, Hannover-Seelze, Germany) through the flow chambers to remove planktonic bacteria. The biofilms were specifically stained live/dead by flushing with a 1:1000 dilution of BacLight staining mix (Life Technologies, Darmstadt, Germany). Syto 9 is a green fluorescent dye that passes bacterial cell membranes by diffusion and intercalates unspecifically into bacterial DNA. Propidium iodide is a red fluorescent dye that, due to its size, cannot pass intact cell membranes. Dye intercalation is only observed in dead cells in which membrane integrity is impaired. The bacteria were stained for 15 min at a flow rate of 100 μL/min in the dark and subsequently examined by confocal laser scanning microscopy (CLSM, Leica-Upright MP microscope connected to a TCS SP2 AOBS scan head, Leica, Wetzlar, Germany). From each specimen, image stacks were acquired at five different positions; centre, right, left, top and bottom, using 10x magnification. The Imaris 3D image processing software (Version 6.2.1, Bitplane, Oxford instruments, Zurich, Switzerland) was used to calculate mean biofilm height.

### Statistical analysis

The experiments were performed five times for each bacterial species, and the mean biofilm height and standard errors were calculated. The mean biofilm heights between independent biological replicates were compared using the univariate ANOVA test with a significance level of 0.05. All statistical analyses were conducted using the software package SPSS (v23.0.0, IBM, Armonk, USA).

## Results

### The flow chamber system

**Fig 2:**
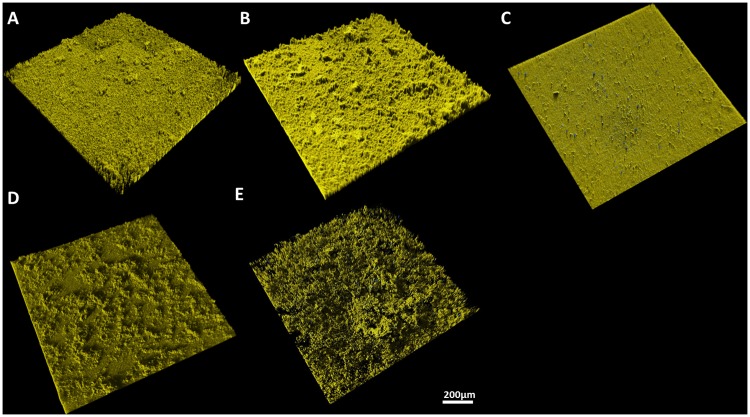
3D reconstruction of biofilms in side view. The cells were stained live/dead and analysed by CLSM. Vital cells are depicted in yellow, viable cells in blue. **A)** *S*. *gordonii*, **B)** *S*. *oralis*, **C)** *S*. *salivarius*, **D)** *P*. *gingivalis*, and **E)** *A*. *actinomycetemcomitans*.

The flow chamber system was successfully manufactured according to the construction plans. An assembly drawing of the chamber is presented in Fig 1A. As test specimen titanium discs were used. To allow for macro-and microscopical specimen observation, the stainless steel cover plate had been manufactured with a circular recess, housing a microscopic coverglass. In Fig 1B, the complete setup of a flow chamber in a continuous flow circuit is depicted. The growth conditions were successfully adjusted under aerobic and anaerobic conditions as seen by the formation of stable, mature biofilms on the titanium specimens (Fig 2).

### Micro- and macroscopic evaluation of biofilm formation

Biofilm formation of *S*. *gordonii*, *S*. *oralis*, *S*. *salivarius*, *P*. *gingivalis* and *A*. *actinomycetemcomitans* was evaluated under constant flow conditions. A confluent biofilm formation was observed for all tested bacterial species. The bacterial cultures in the bioreactor showed normal growth behavior during the experiment and the resulting biofilms were structurally intact and composed of predominantly vital cells (Fig 2A–2E). For all species, biofilm formation was highly reproducible between independent experiments. However, the observed biofilm morphologies were unique to the individual species. In detail, *S*. *gordonii* (A) and *S*. *salivarius* (C) biofilms showed relatively homogenous surface pattern interspersed with a few tower-like structures. *S*. *oralis* (B) biofilms demonstrated an uneven surface pattern with numerous tower-like structures. The *P*. *gingivalis* (D) biofilm showed a rough biofilm surface with marcocolonies. The biofilm of *A*. *actinomycetemcomitans* (E) demonstrated an open and loose microbial biofilm structure.

Throughout all experiments, the biofilms exhibited high structural stability and no visible detachment was detected during sample preparation for microscopic analysis.

### Calculation of mean biofilm height

**Fig 3:**
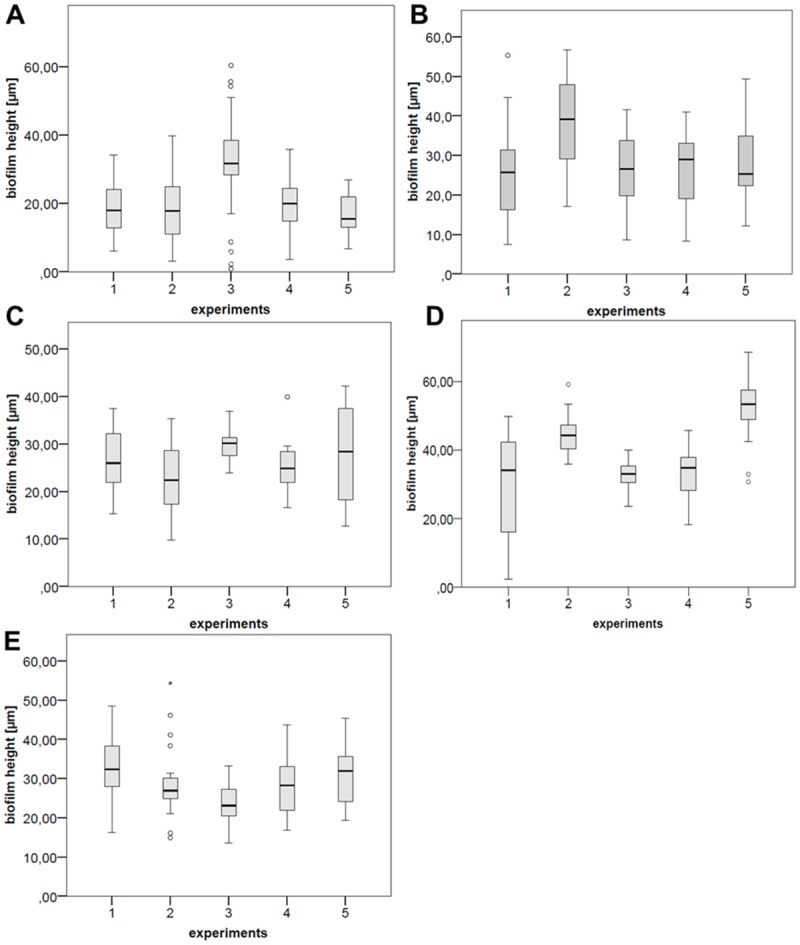
Biofilm heights on titanium substrata in the flow chamber system. In each diagram, the mean biofilm heights for five independent experiments are shown. **A** = *S*. *gordonii*, ***B*** = *S*. *oralis*, **C** = *S*. *salivarius*, **D** = *P*. *gingivalis*, and **E** = *A*. *actinomycetemcomitans*.

Mean biofilm heights were determined for the five bacterial species tested. For each species, the reproducibility of biofilm formation between the independent experiments was statistically significant (Fig 3). The greatest mean biofilm height was observed for *P*. *gingivalis* with 38.85 μm (*p* = *5*.*2 x 10*^*−13*^), followed by *A*. *actinomycetemcomitans* with 28.9 μm (*p* = *0*.*01*), *S*. *oralis* with 28.5 μm (*p* = *0*.*003*), *S*. *salivarius* with 26.5 μm (*p* = *0*.*008*), and *S*. *gordonii* with 21.1 μm (*p* = *0*.*000009*).

## Discussion

Many flow chamber systems have been developed within recent decades to analyse biofilm formation and dynamics in a fluid system. Our flow chamber system was optimized for the experimental investigation of dental relevant bacteria on the implant material titanium, as this is an important approach to prevent implant failure due to biofilm-associated infections. Within the presented flow chamber system, the replacement of the titanium disc with other disc-shaped sample materials of interest is applicable without time-consuming reconstruction measures. The complete flow chamber system is reusable and fully autoclavable. The cultivation setup can easily be modified according to the optimal growth conditions of the respective bacterial species under aerobic as well as anaerobic conditions.

We focused on a group of bacteria, consisting of oral commensal and periodontopathogenic bacteria: *S*. *gordonii*, *S*. *oralis*, *S*. *salivarius*, *P*. *gingivalis*, and *A*. *actinomycetemcomitans*. Previous studies have mostly described biofilm formation under static test conditions [52, 53], but *in vivo* the flow has great influence on the of the biofilm behavior. Therefore, fluid systems have been established to mimic the physiological flow conditions within the oral cavity [54]. To achieve a realistic experimental setup, we chose a flow speed of 100 μL/min, as it is described for the natural saliva flow in the hibernation mode [55–57]. The bacterial suspension was pumped continuously over the test specimens as in the oral cavity bacteria-contaminated saliva is permanently flooding the implant.

All bacterial species exhibited reproducible and homogenous growth behavior under the given flow conditions, as confirmed by 3D biofilm reconstruction from CLSM image stacks and statistical analyzes.

In contrast to the flow chamber systems of Weiger et al., Hauser-Gerspach et al., Meier et al., and Dia*z* et al., our test procedure allows direct investigation of biofilm formation for five bacterial species [56, 58–64]. The test specimens were not removed from the chambers for microscopic observation, so that detachment effects were minimised and biofilm quantification was highly precise. Only a few studies have analysed biofilm development for oral health relevant bacteria in a flow system [56, 58–64]. To the best of our knowledge, no other study has focused on the described group of bacteria for microbial adhesion testing in one flow chamber system.

The biofilm morphologies of the applied bacterial species have already been in other studies: In accordance to the study of Diaz et al., a homogeneously flat biofilm morphology with a few tower-shaped structures was observed for *S*. *gordonii*. However, contrasting to our study the bacteria were visualized by fluorescent in situ hybridization (FISH) [56].

For *S*. *oralis* Paramonova et al. designed a flow chamber system to analyse the influence of shear stress on biofilm formation [61]. The biofilm height of *S*. *oralis* was enhanced with increasing shear stress. The observed biofilm morphology of *S*. *oralis* showed a typical tower-like biofilm with a rough surface comparable to our study.

The *S*. *salivarius* biofilm morphology was homogenously flat, as also shown in the study of Gashti et al. [65]. However, they used microfluidic flow chambers, and focused primarily on the influence of pH on biofilm formation.

Davey et al. designed their flow chamber model for *P*. *gingivalis* according to Christensen et al. [64, 66]. They also observed a rough surface morphology with macrocolonies within the biofilm. Analogously to our experiments, the biofilm was grown under anaerobic conditions. However, the mean biofilm height was about five times higher compared to our study. These findings can be attributed to the different flow chamber design and the differing cultivation conditions. Davey et al. grew biofilms for 96 h compared to 48 h in our experiment [64]. Additionally, Davey et al. analyzed the living fraction of the biofilm by staining with SYTO9.

The biofilm morphology of *A*. *actinomycetemcomitans* showed an open and soft microbial architecture on the titanium substratum. The same findings were described by Sliepen et al. [63]. In contrast to our method, they tagged *A*. *actinomycetemcomitans* with a green fluorescent protein to analyze the biofilm formation by CLSM. However, it cannot be completely ruled out that this genetic modification may have influenced the adhesion behavior and biofilm formation.

Finally, in the present study, the reproducibility of the bacterial biofilm height was given in all our experiments.

Biofilm formation takes place throughout the whole oral cavity. However, daily oral hygiene measures reduce the amount of attached biofilms in the oral cavity. Especially at the interfaces gum/tooth or gum/implant bacteria begin to accumulate and form biofilms. If not removed, biofilms cause swelling and detachment of the gums from teeth or implants. Biofilms further proliferate in the formed periodontal pockets. As these are not isolated compartments but are connected to the oral cavity, biofilms are exposed to a low flow environment rather than static conditions. Therefore, the described flow chamber model approximates the *in vivo* situation, which is crucial for evaluation of surfaces intended to be used in the oral cavity. In conclusion, the here developed flow chamber system, in combination with CLSM-based biofilm quantification, proved to be a reliable instrument for the analysis of biofilm height and the formation of bacterial biofilms that are relevant in dentistry. Under the given experimental setting, the flow chambers can be used for evaluation of bacterial colonisation behavior of implant materials for the oral cavity. In further studies, the system will be optimised for studies of the formation oral multispecies biofilms, which is closer to the actual situation in the human mouth. Other interesting aspects will be the investigation of the influence of different flow velocities, nutrient concentrations and substrata on the biofilm formation.

## Supporting information

S1 File Supporting information. Complementary information of all biofilm height data in μm are accessible. The tables are subdivided in live and dead bacterial biofilm populations.
(DOCX)
