# Supplementary material for: Development of a flow chamber system for the reproducible in vitro analysis of biofilm formation on implant materials
Source: PLoS One. 2017 Feb 10;12(2):e0172095. doi: 10.1371/journal.pone.0172095 (PMC5302373; doi:10.1371/journal.pone.0172095)
Supplement: S1 File — Complementary information of all biofilm height data in μm are accessible. The tables are subdivided in live and dead bacterial biofilm populations. (DOCX) [file pone.0172095.s001.docx]

| ***Streptococcus gordonii*** | | | |  | |  | |  |  | | |  |  | | | | |  |  |
| --- | --- | --- | --- | --- | --- | --- | --- | --- | --- | --- | --- | --- | --- | --- | --- | --- | --- | --- | --- |
|  |  | **Biofilm heights in µm LIVE** | | | |  | |  | **Biofilm heights in µm DEAD** | | | | | | |  |  | | |
| Experi. |  | Middle | Top | | Bottom | Right | Left | | Middel | Top | Bottom | | | Right | Left | | | |  |
| 1 | Chamber 1 | 9.6 | 10.1 | | 16.2 | 12.9 | 22.6 | | 1,12E-07 | 6,11E-08 | 1,69E-02 | | | 5,78E-07 | 7,57E-08 | | |  |  |
|  | Chamber 2 | 26.4 | 12.4 | | 20.7 | 24.1 | 28.4 | | 2,25E-07 | 4,37E-08 | 9,14E-02 | | | 2,94E-02 | 1,92E-01 | | |  |  |
|  | Chamber 3 | 16.9 | 12.6 | | 18.6 | 18.0 | 13.1 | | 7,43E-04 | 3,16E-07 | 4,10E-07 | | | 2,61E-07 | 1,34E-02 | | |  |  |
|  | Chamber 4 | 19.8 | 9.29 | | 20.8 | 12.9 | 6.11 | | 3,24E-07 | 3,88E-08 | 4,45E-02 | | | 9,21E02 | 5,93E-07 | | |  |  |
|  | Chamber 5 | 28.0 | 29.9 | | 27.5 | 34.1 | 12.9 | | 4,21E-01 | 4,81E-01 | 3,07E-02 | | | 3,11E-01 | 3,94E-01 | | |  |  |
| 2 | Chamber 1 | 24.3 | 27.9 | | 35.9 | 28.7 | 32.4 | | 1,67E-02 | 6,06E-06 | 1,56E-07 | | | 2,62E-07 | 6,38E-07 | | |  |  |
|  | Chamber 2 | 11.1 | 11.9 | | 14.1 | 11.4 | 8.21 | | 4,03E-02 | 3,57E-01 | 1,65E-07 | | | 6,43E-07 | 8,65E-08 | | |  |  |
|  | Chamber 3 | 24.8 | 17.8 | | 18.6 | 19.8 | 26.9 | | 2,25E-07 | 3,24E-07 | 4,76E-07 | | | 7,14E-02 | 4,36E-01 | | |  |  |
|  | Chamber 4 | 7.5 | 10.8 | | 19.4 | 23.3 | 13.9 | | 1,24E+00 | 1,38E-01 | 1,11E-01 | | | 1,54E-01 | 1,19E-01 | | |  |  |
|  | Chamber 5 | 39.8 | 8.86 | | 11.6 | 5.74 | 3.06 | | 5,71E-02 | 1,66E-02 | 5,29E-01 | | | 1,39E-02 | 1,26E-07 | | |  |  |
| 3 | Chamber 1 | 48.8 | 31.3 | | 55.6 | 34.9 | 54.4 | | 1,12E-07 | 2,61E-07 | 1,27E+00 | | | 1,43E+00 | 3,25E+00 | | |  |  |
|  | Chamber 2 | 36.8 | 33.4 | | 34.4 | 41.9 | 30.5 | | 1,95E-01 | 7,11E-07 | 3,16E+00 | | | 1,35E+01 | 9,71E+00 | | |  |  |
|  | Chamber 3 | 5.84 | 28.4 | | 2.24 | 0.87 | 17.1 | | 8,86E-02 | 1,16E+00 | 6,26E-07 | | | 3,02E-07 | 1,14E-01 | | |  |  |
|  | Chamber 4 | 8.71 | 31.6 | | 30.5 | 17.0 | 31.4 | | 3,02E-01 | 3,43E-02 | 3,27E-01 | | | 1,09E-01 | 2,11E+00 | | |  |  |
|  | Chamber 5 | 60.3 | 34.0 | | 38.4 | 50.9 | 28.7 | | 4,51E-02 | 1,30E-01 | 6,30E+00 | | | 5,59E+00 | 4,03E+00 | | |  |  |
| 4 | Chamber 1 | 23.0 | 6.49 | | 9.14 | 5.3 | 16.4 | | 1,95E-01 | 7,11E-07 | 8,29E-03 | | | 2,55E-07 | 4,77E-02 | | |  |  |
|  | Chamber 2 | 19.8 | 35.8 | | 20.0 | 13.6 | 29.3 | | 8,86E-02 | 1,16E+00 | 2,05E+00 | | | 3,95E-02 | 3,04E+00 | | |  |  |
|  | Chamber 3 | 31.6 | 26.1 | | 35.8 | 3.61 | 21.5 | | 3,02E-01 | 3,43E-02 | 2,55E-01 | | | 2,64E+00 | 1,72E-01 | | |  |  |
|  | Chamber 4 | 18.4 | 16.0 | | 16.1 | 22.6 | 22.7 | | 4,51E-02 | 1,30E-01 | 5,33E-02 | | | 4,66E-01 | 3,95E-01 | | |  |  |
| 5 | Chamber 1 | 8.5 | 15.9 | | 25.4 | 15.0 | 21.1 | | 2,51E-06 | 1,23E-01 | 1,12E-02 | | | 1,03E-02 | 3,36E-01 | | |  |  |
|  | Chamber 2 | 10.2 | 22.6 | | 11.4 | 13.6 | 6.79 | | 2,96E-06 | 1,64E-02 | 3,50E-06 | | | 4,47E-06 | 1,18E-01 | | |  |  |
|  | Chamber 3 | 16.9 | 16.7 | | 17.1 | 14.7 | 13.1 | | 2,20E-02 | 6,69E-02 | 1,21E-01 | | | 8,07E-02 | 1,81E-03 | | |  |  |
|  | Chamber 4 | 14.3 | 26.9 | | 23.9 | 26.0 | 12.9 | | 1,29E-01 | 1,98E-01 | 7,90E-02 | | | 2,62E-06 | 3,85E-02 | | |  |  |

| ***Streptococcus oralis*** | | |  |  |  |  |  |  | |  | |  | |  |
| --- | --- | --- | --- | --- | --- | --- | --- | --- | --- | --- | --- | --- | --- | --- |
|  |  | **Biofilm heights in µm LIVE** | |  |  |  | **Biofilm heights in µm DEAD** | | | | | | |  |
| Experi. |  | Middle | Top | Bottom | Right | Left | Middel | | Top | | Bottom | | Right | Left |
| 1 | Chamber 1 | 37.9 | 35.7 | 40.1 | 44.6 | 26.0 | 0.46 | | 1.04 | | 1.24 | | 2.20 | 0.43 |
|  | Chamber 2 | 27.6 | 25.7 | 31.3 | 23.2 | 29.6 | 0.45 | | 2.18 | | 1.53 | | 1.24 | 1.84 |
|  | Chamber 3 | 18.4 | 22.5 | 18.5 | 18.4 | 16.0 | 0.60 | | 0.71 | | 1.35 | | 0.52 | 0.79 |
|  | Chamber 4 | 13.6 | 16.0 | 16.3 | 16.1 | 7.42 | 0.08 | | 0.01 | | 0.01 | | 0.06 | 0.03 |
|  | Chamber 5 | 31.6 | 27.8 | 55.3 | 15.5 | 29.6 | 1.51 | | 1.50 | | 6.71 | | 0.57 | 1.02 |
| 2 | Chamber 1 | 19.1 | 17.1 | 23.1 | 24.6 | 28.1 | 1.16 | | 1.57 | | 2.19 | | 2.22 | 9.29 |
|  | Chamber 2 | 48.7 | 37.1 | 51.1 | 38.6 | 47.6 | 5.19 | | 5.79 | | 0.83 | | 6.25 | 5.36 |
|  | Chamber 3 | 56.6 | 44.6 | 48.1 | 39.6 | 42.2 | 13.86 | | 4.24 | | 6.65 | | 6.52 | 8.50 |
|  | Chamber 4 | 37.2 | 43.1 | 49.6 | 30.2 | 37.4 | 7.0 | | 5.06 | | 6.82 | | 4.54 | 4.94 |
| 3 | Chamber 1 | 8.64 | 15.7 | 14.2 | 27.8 | 17.9 | 0.23 | | 0.65 | | 1.32 | | 2.25 | 0.12 |
|  | Chamber 2 | 20.6 | 36.9 | 41.6 | 33.6 | 33.8 | 0.12 | | 2.96 | | 3.83 | | 1.70 | 0.99 |
|  | Chamber 3 | 34.6 | 21.1 | 31.6 | 29.0 | 26.6 | 0.55 | | 2.0 | | 0.45 | | 3.74 | 1.77 |
|  | Chamber 4 | 26.6 | 25.6 | 20.9 | 37.2 | 19.0 | 0.59 | | 3.12 | | 0.98 | | 2.77 | 0.21 |
| 4 | Chamber 1 | 11.8 | 31.2 | 11.1 | 21.5 | 8.26 | 0.09 | | 1.84 | | 0.20 | | 0.16 | 0.001 |
|  | Chamber 2 | 39.9 | 19.0 | 39.9 | 22.4 | 27.7 | 0.97 | | 0.50 | | 2.72 | | 0.003 | 0.002 |
|  | Chamber 3 | 31.6 | 34.0 | 35.7 | 41.0 | 31.0 | 3.20 | | 3.16 | | 1.14 | | 1.84 | 2.87 |
|  | Chamber 4 | 32.7 | 31.0 | 29.0 | 33.0 | 34.0 | 1.27 | | 1.95 | | 1.01 | | 4.26 | 2.89 |
|  | Chamber 5 | 24.5 | 20.7 | 18.6 | 12.6 | 17 | 1.50 | | 0.01 | | 1.14 | | 0.38 | 0.06 |
| 5 | Chamber 1 | 14.8 | 28.9 | 22.3 | 23.9 | 38.1 | 5.15 | | 0.81 | | 1.70 | | 8.29 | 11.00 |
|  | Chamber 2 | 29.9 | 25.3 | 35.0 | 34.9 | 49.3 | 8.07 | | 3.54 | | 5.50 | | 12.50 | 13.00 |
|  | Chamber 3 | 17.1 | 21.6 | 22.1 | 22.6 | 41.3 | 2.97 | | 5,17 | | 1.20 | | 4.56 | 12.40 |
|  | Chamber 4 | 38.4 | 28.2 | 12.1 | 22,4 | 41.3 | 4.71 | | 6.59 | | 0.47 | | 1.56 | 5.42 |
|  | Chamber 5 | 27.4 | 25.3 | 25.3 | 24.7 | 19.1 | 13.28 | | 9.57 | | 11.20 | | 9.50 | 5.67 |

| ***Streptococcus salivarius*** | | | | | | | | | | | | |  | | | | |  |
| --- | --- | --- | --- | --- | --- | --- | --- | --- | --- | --- | --- | --- | --- | --- | --- | --- | --- | --- |
|  |  | **Biofilm heights in µm LIVE** | | |  |  |  | **Biofilm heights in µm DEAD** | | |  | | |  |  |  | | |
| Experi. |  | Middle | Top | Bottom | | Right | Left | Middel | Top | Bottom | Right | | Left | | | | |  |
| 1 | Chamber 1 | 24.6 | 22.1 | 18.9 | | 27.1 | 24.4 | 0.009 | 0.012 | 0.000002 | 0.707 | | 0.000005 | | | | |  |
|  | Chamber 2 | 18.4 | 21.6 | 15.3 | | 24.4 | 21.2 | 0.000003 | 0.149 | 0.000005 | 0.015 | | 0.032 | | | | |  |
|  | Chamber 3 | 37.5 | 25.6 | 28.4 | | 28.6 | 26.3 | 0.076 | 0.055 | 0.644 | 3.457 | | 0.086 | | | |  |  |
|  | Chamber 4 | 32.4 | 36.4 | 34.4 | | 32.2 | 32.1 | 0.204 | 00081 | 0.500 | 0.071 | 0.013 | | | | | |  |
| 2 | Chamber 1 | 17.8 | 13.1 | 22.7 | | 21.7 | 10.1 | 0.043 | 0.007 | 0.138 | 0.000006 | 0.000001 | | | | | |  |
|  | Chamber 2 | 28.9 | 10.4 | 20.4 | | 14.6 | 27.9 | 0.000004 | 0,024 | 0.031 | 0.00778 | 0.000001 | | | | | |  |
|  | Chamber 3 | 26.7 | 33.6 | 33.5 | | 24.2 | 28.6 | 0.193 | 0.000002 | 0.015 | 0.000004 | 0.000001 | | | | | |  |
|  | Chamber 4 | 35.3 | 19.3 | 33.2 | | 33.9 | 19.3 | 0.013 | 0.530 | 0.000001 | 0.000002 | 0.007 | | | | | |  |
|  | Chamber 5 | 22.4 | 17.3 | 9.71 | | 24.1 | 16.0 | 0.000002 | 0.557 | 0.000001 | 0.809 | 0.000001 | | | | | |  |
| 3 | Chamber 1 | 31.1 | 30.2 | 30.0 | | 31.6 | 30.3 | 0.629 | 2.464 | 0.300 | 0.176 | 3.507 | | | | | |  |
|  | Chamber 2 | 26.5 | 24.8 | 36.8 | | 24.3 | 23.9 | 0.640 | 2.343 | 3.464 | 1.607 | 0.807 | | | | | |  |
|  | Chamber 3 | 33.8 | 30.8 | 24.9 | | 29.9 | 30.8 | 0.096 | 3.450 | 1.000 | 0.829 | 0.032 | | | | | |  |
|  | Chamber 4 | 29.9 | 29.8 | 31.6 | | 28.6 | 36.1 | 2.000 | 3.650 | 0.886 | 0.058 | 1.514 | | | | | |  |
| 4 | Chamber 1 | 27.6 | 21.4 | 25.9 | | 29.2 | 25.5 | 0.000006 | 0.008 | 0.017 | 4.850 | 0.106 | | | | | |  |
|  | Chamber 2 | 29.5 | 39.9 | 23.3 | | 24.1 | 23.1 | 0.250 | 0.030 | 10.143 | 0.485 | 9.857 | | | | | |  |
|  | Chamber 3 | 20.9 | 22.1 | 25.9 | | 21.7 | 16.6 | 0.065 | 0.000002 | 0.516 | 0.294 | 0.081 | | | | | |  |
|  | Chamber 4 | 29.5 | 19.7 | 22.1 | | 29.4 | 25.6 | 0.316 | 0.521 | 0.176 | 0.218 | 0.087 | | | | | |  |
| 5 | Chamber 1 | 28.4 | 29.6 | 18.9 | | 24.1 | 36.4 | 0.197 | 0.729 | 0,088 | 0.5987 | 0.036 | | | | | |  |
|  | Chamber 2 | 40.0 | 42.2 | 38.5 | | 32.9 | 15.5 | 0.015 | 0.001 | 0.126 | 0.018 | 0.014 | | | | | |  |
|  | Chamber 3 | 24.7 | 42.2 | 12.7 | | 27.4 | 34.8 | 0.119 | 0.002 | 0.033 | 0.069 | 0.648 | | | | | |  |
|  | Chamber 4 | 28.4 | 15.0 | 40.9 | | 17.6 | 17.6 | 0.012 | 0.0001 | 3.043 | 0.160 | 0.005 | | | | | |  |

| ***Porphyromonas gingivalis*** | | | | | | | | | | | | | | | | | | | | |
| --- | --- | --- | --- | --- | --- | --- | --- | --- | --- | --- | --- | --- | --- | --- | --- | --- | --- | --- | --- | --- |
|  |  | | | **Biofilm heights in µm LIVE** | | | | | | | | **Biofilm heights in µm DEAD** | | | | | | | | |
| Experi. | |  | Middle | | Top | | Bottom | Right | | Left | Middel | | Top | Bottom | | | Right | | Left |  |
| 1 | Chamber 1 | | | 22.64 | | 2.4 | 23.5 | | 16.4 | 25.57 | 2.40 | | 4.12 | | 4.43 | 4.43 | | 2.82 | | |
|  | Chamber 2 | | | 49.78 | | 7.42 | 42.21 | | 42.21 | 45.28 | 7.42 | | 19.57 | | 10.0 | 10.0 | | 8.50 | | |
|  | Chamber 3 | | | 39.64 | | 6.6 | 34 | | 34 | 43.42 | 6.60 | | 12.85 | | 5.87 | 5.81 | | 10.42 | | |
| 2 | Chamber 1 | | | 45.0 | | 45.0 | 36.1 | | 39.6 | 44.4 | 3.36 | | 3.20 | | 1.26 | 1.84 | | 4.06 | | |
|  | Chamber 2 | | | 35.8 | | 35.8 | 37.5 | | 49.5 | 59.1 | 1.53 | | 1.76 | | 2.22 | 2.19 | | 6.29 | | |
|  | Chamber 3 | | | 41.1 | | 41.1 | 50.6 | | 44 | 53.1 | 2.26 | | 3.71 | | 1.79 | 1.51 | | 3.19 | | |
|  | Chamber 4 | | | 44.2 | | 44.2 | 44.5 | | 53.3 | 43.6 | 9.93 | | 2.34 | | 1.51 | 2.83 | | 2.11 | | |
| 3 | Chamber 1 | | | 37.6 | | 34.2 | 23.5 | | 33.3 | 32.1 | 0.36 | | 0.93 | | 0.08 | 0.32 | | 0.29 | | |
|  | Chamber 2 | | | 32.7 | | 26.6 | 29.4 | | 35.2 | 40 | 0.27 | | 0.026 | | 0.29 | 0.64 | | 0.07 | | |
|  | Chamber 3 | | | 25.4 | | 35,4 | 31.5 | | 29.1 | 33.9 | 0.06 | | 0.3 | | 0.16 | 0.4 | | 0.08 | | |
|  | Chamber 4 | | | 37.34 | | 31,6 | 32.0 | | 34.9 | 39.1 | 0.25 | | 0.27 | | 0.11 | 0.22 | | 0.27 | | |
| 4 | Chamber 1 | | | 38.4 | | 41,9 | 42.1 | | 38.5 | 32.7 | 0.26 | | 0.24 | | 3.68 | 2.64 | | 2.64 | | |
|  | Chamber 2 | | | 45.6 | | 35,1 | 36.6 | | 25.7 | 18.3 | 0.32 | | 2.64 | | 2.60 | 0.18 | | 0.18 | | |
|  | Chamber 3 | | | 26.1 | | 37,3 | 30.2 | | 32.7 | 33.8 | 4.25 | | 2.64 | | 0.26 | 1.83 | | 0.018 | | |
|  | Chamber 4 | | | 34.9 | | 26 | 22.9 | | 34.6 | 36.6 | 4.90 | | 0.08 | | 0.39 | 0.09 | | 2.64 | | |
| 5 | Chamber 1 | | | 49.2 | | 52,9 | 52.2 | | 42.5 | 48.6 | 2.00 | | 1.27 | | 9.20 | 5.5 | | 0.9 | | |
|  | Chamber 2 | | | 57.5 | | 32,9 | 62.2 | | 57.3 | 56.4 | 2.3 | | 2.95 | | 2.3 | 1.6 | | 1.6 | | |
|  | Chamber 3 | | | 49.8 | | 63,6 | 68.5 | | 45 | 30.7 | 1.9 | | 20.7 | | 7.1 | 1.6 | | 13.5 | | |
|  | Chamber 4 | | | 57.2 | | 53,6 | 55 | | 53.2 | 63.1 | 1.93 | | 3.2 | | 6.8 | 1.4 | | 21.14 | | |

| ***Aggregatibacter actinomycetemcomitans*** | | | | |  |  |  |  |  |  |  |  |
| --- | --- | --- | --- | --- | --- | --- | --- | --- | --- | --- | --- | --- |
|  |  | **Biofilm heights in µm LIVE** | |  | |  |  | **Biofilm heights in µm DEAD** | | | | |
| Experi. |  | Middle | Top | Bottom | | Right | Left | Middel | Top | Bottom | Right | Left |
| 1 | Chamber 1 | 34.3 | 30.1 | 31.9 | | 16.2 | 29.7 | 2.690 | 2.110 | 0.929 | 0.237 | 0.978 |
|  | Chamber 2 | 17.9 | 32.7 | 34.9 | | 43.2 | 43.8 | 0.263 | 1.340 | 2.450 | 3.350 | 6.150 |
|  | Chamber 3 | 48.5 | 37.9 | 37.6 | | 38.6 | 28.3 | 6.860 | 3.260 | 1.820 | 1.840 | 2.200 |
|  | Chamber 4 | 30.4 | 21.6 | 27.7 | | 16.4 | 44.1 | 3.290 | 1.740 | 2.180 | 0.526 | 4.200 |
| 2 | Chamber 1 | 24.9 | 21.1 | 25.9 | | 14.9 | 16.1 | 0.140 | 0.520 | 0.130 | 0.065 | 0.100 |
|  | Chamber 2 | 26.9 | 26.9 | 28.4 | | 27.4 | 38.4 | 0.040 | 0.400 | 0.010 | 0.130 | 0.040 |
|  | Chamber 3 | 46.1 | 54.3 | 41.1 | | 25.1 | 24.7 | 0.400 | 0.890 | 0.340 | 0.130 | 0.200 |
|  | Chamber 4 | 22.9 | 31.4 | 38.6 | | 25.7 | 28.9 | 0.030 | 0.110 | 0.600 | 0.068 | 0.190 |
| 3 | Chamber 1 | 13.9 | 19.1 | 21.1 | | 26.3 | 32.0 | 0.025 | 0.038 | 0.475 | 0.319 | 0.175 |
|  | Chamber 2 | 29.9 | 20.3 | 20.6 | | 21.4 | 21.4 | 0.024 | 0.028 | 0.351 | 0.165 | 0.039 |
|  | Chamber 3 | 13.9 | 25.7 | 13.5 | | 33.2 | 28.2 | 0.035 | 0.095 | 0.057 | 0.0001 | 0.079 |
|  | Chamber 4 | 24.1 | 22.2 | 25.1 | | 24.0 | 32.0 | 0.051 | 0.115 | 0.103 | 0.358 | 0.081 |
| 4 | Chamber 1 | 21.4 | 18.9 | 32.4 | | 37.1 | 23.1 | 0.114 | 0.021 | 0.030 | 0.037 | 0.064 |
|  | Chamber 2 | 22.3 | 19.6 | 33.6 | | 31.9 | 43.6 | 0.118 | 0.026 | 0.185 | 0.156 | 1.090 |
|  | Chamber 3 | 30.4 | 27.3 | 31.4 | | 27.0 | 24.6 | 0.110 | 0.105 | 0.291 | 0.0154 | 0.174 |
|  | Chamber 4 | 29.1 | 16.8 | 40.6 | | 42.0 | 18.4 | 0.586 | 0.085 | 0.221 | 1.840 | 0.055 |
| 5 | Chamber 1 | 34.5 | 21.3 | 31.6 | | 27.4 | 31.9 | 1.370 | 1.250 | 0.393 | 0.409 | 1.400 |
|  | Chamber 2 | 29.5 | 41.3 | 24.4 | | 19.4 | 22.3 | 1.640 | 1.020 | 1.140 | 0.329 | 0.091 |
|  | Chamber 3 | 35.6 | 21.2 | 38.9 | | 31.9 | 33.9 | 0.368 | 0.547 | 0.914 | 0.979 | 0.714 |
|  | Chamber 4 | 45.5 | 32.1 | 23.9 | | 35.6 | 39.8 | 0.921 | 0.426 | 1.040 | 0.130 | 0.663 |
